# Childhood adversity as a risk for cancer: findings from the 1958 British birth cohort study

**DOI:** 10.1186/1471-2458-13-767

**Published:** 2013-08-19

**Authors:** Michelle Kelly-Irving, Benoit Lepage, Dominique Dedieu, Rebecca Lacey, Noriko Cable, Melanie Bartley, David Blane, Pascale Grosclaude, Thierry Lang, Cyrille Delpierre

**Affiliations:** 1 INSERM, U1027, Toulouse F-31300, France; 2 Université Toulouse III Paul-Sabatier, UMR1027, Toulouse F-31300, France; 3 CHU Toulouse, Hôpital Purpan, Département, Toulouse F-31300, France; 4 Department of epidemiology and public health, University College London, London, UK; 5 Department of Primary Care and Public Health, Imperial College London, London, UK; 6 Institut Claudus Regaud, Toulouse F-31300, France

**Keywords:** (MeSH): Social medicine, Cancer, Psychosocial factors, Cohort studies, Psychological stress, Life course

## Abstract

**Background:**

To analyse whether Adverse Childhood Experiences (ACE) are associated with an increased risk of cancer.

**Methods:**

The National child development study (NCDS) is a prospective birth cohort study with data collected over 50 years. The NCDS included all live births during one week in 1958 (n = 18558) in Great Britain. Self-reported cancer incidence was based on 444 participants reporting having had cancer at some point and 5694 reporting never having cancer. ACE was measured using reports of: 1) child in care, 2) physical neglect, 3) child’s or family’s contact with the prison service, 4) parental separation due to divorce, death or other, 5) family experience of mental illness & 6) family experience of substance abuse. The resulting variable had three categories, no ACEs/ one ACE/ 2 + ACEs and was used to test for a relationship with cancer. Information on socioeconomic characteristics, pregnancy and birth were extracted as potential confounders. Information on adult health behaviours, socioeconomic environment, psychological state and age at first pregnancy were added to the models. Multivariate models were run using multiply-imputed data to account for missing data in the cohort.

**Results:**

The odds of having a cancer before 50 y among women increased twofold for those who had 2+ ACEs versus those with no ACEs, after adjusting for adult factors and early life confounders (OR: 2.1, 95% CI: 1.42-3.21, p < 0.001).

**Conclusion:**

These findings suggest that cancer risk may be influenced by exposure to stressful conditions and events early on in life. This is potentially important in furthering our understanding of cancer aetiology, and consequently in redirecting scientific research and developing appropriate prevention policies.

## Background

Strong evidence for socio-economic differences in cancer survival and mortality has been shown for many cancers and across populations [1]. In 2004, cancer was among the leading four causes of death in high income countries [2,3], and its increase is typically associated with the third stage of epidemiological transition [4]. Cancer prevalence and mortality is likely to become increasingly predominant in high-income countries and by consequence so is their contribution to health inequalities.

The causes of cancer are complex and multifactorial occurring over a lengthy time. Explanations for mechanisms linking adverse events early in life and cancer have been provided from animal models [5,6]. In humans, early life exposure to Adverse Childhood Experiences (ACE), like trauma, abuse or maltreatment in childhood has been linked both ‘indirectly’, through tobacco and alcohol use or via ‘direct’ associations [7] to alterations of the brain structure and neurobiological stress-response systems which in turn have consequences for health and emotional well-being [8]. Studies have described associations between retrospectively collected ACE and health outcomes such as liver disease [9], ischaemic heart disease [10], obesity [11,12], perceived health [13] and psychopathology [14] as well as premature mortality [15,16]. Regarding cancer, data are sparser and inconsistent [7,12,17]. One of the reasons for this inconsistency is that the ACE measure used is usually self-reported by adults asked about trauma and adversity they may have experienced during childhood. Such questions are inevitably vulnerable to various forms of recall bias [18] one example being that individuals’ memories of the past may be influenced by their later health [19].

In the present study we aim to determine if exposure to ACE, determined using prospectively collected data, is linked with the onset of cancer in adulthood. The causes of cancers are indeed diverse, however our aim here is to understand the development of susceptibility to this disease at its earliest phase, at the common roots of all cancers [20]. To do this we take a lifecourse approach, considering the whole life span. We hypothesise that the physiological embedding of stress induced by adversity in childhood is linked with biological susceptibility as well as with the development of stress-reducing health behaviours favouring the development of cancers in adult life [21,22]. The objective of this study is to examine the relationship between psychosocial adversity in childhood and cancer after controlling for the effects of material disadvantage, health behaviours and education level using a large prospective cohort study.

## Methods

Data are from the 1958 National Child Development Study (NCDS) which included all live births during one week in 1958 (n = 18558) in Great Britain. Subsequent data collections (sweeps) were carried out on cohort members aged 7, 11, 16, 23, 33, 42, 46 and 50. At the last sweep, carried out in 2008, 9790 individuals participated in the self-reported questionnaire and face to face interview, representing 53% of the original sample. The NCDS has been described in detail elsewhere [23].

### Ethics & data access

Written informed consent was obtained from the parents for childhood measurements and ethical approval for the adult data collection was obtained from the National Research Ethics Advisory Panel. NCDS data are open access datasets available to non-profit research organisations.

### Cancer in the cohort

The outcome variable of interest in this study was self-reported cancer between the age of 33 and 50. We did not use information available on cancer at the age of 23, as we were interested in controlling for health behaviours at this age. Two main types of variables were used across the data sweeps. At ages 46 and 50 respondents were asked to report any medical conditions among which cancers can be identified. At ages 33 and 42 respondents were asked to report if they had ‘ever had cancer’. The outcome variable ‘cancer’ constructed for the purpose of this study was categorised as follows: ‘yes’ corresponds to individuals who ever reported having had cancer at ages 33, 42, 46 or 50 (n = 444); ‘no’ corresponds to individuals who explicitly reported not having a cancer at age 33 and 42, and who did not have an ICD coded cancer among their reported medical conditions at the ages of 46 and 50 (n = 5694); ‘missing’ corresponds to individuals who did not respond to any questions on cancer, and respondents who occasionally responded ‘no’ or who were missing. These are individuals who we could not exclude as having had a cancer based on their self-report (n = 11943). Based on this conservative definition respondents had to actively respond ‘yes’ to having had a cancer or report a cancer as a medical condition. Conversely they had to consistently respond ‘no’ to questions on cancer and not report cancer as a medical condition. All other cases were defined as ‘missing’. Further details on how cancer was coded in the cohort are available from the authors.

### Adverse childhood experiences (ACE)

There are many ways in which adversity can be conceptualised [24-27]. We have attempted to construct a theoretical framework prior to extracting any data, in order to create a measurement with a robust content validity. We have identified ACE as a set of traumatic and stressful psychosocial conditions that are out of the child’s control, that tend to co-occur and often persist over time [12,14,24,27]. We have restricted ACE to intra-familial events or conditions in the child’s immediate environment causing chronic stress responses. In our definition ACE is distinguished from events or conditions linked to the socioeconomic and material environment.

Information was extracted from the study via variables collected at age 7, 11, 16 from questions posed to the child’s parent or their teacher. Sources of adversity were divided into six categories:

1. Child in care: child has ever been in public/ voluntary care services or foster care at age 7, 11 or 16.

2. Physical neglect: child appears undernourished/ dirty aged 7 or 11, information collected from the response from child’s teacher to the Bristol Social Adjustment Guide.

*Household dysfunction*, as described by Felitti et al. [12], is a dimension of adversity consisting of four categories each contributing to the score:

3. Offenders: The child lived in a household where a family member was in prison or on probation (age 11 y) or is in contact with probation service at 7 or 11 y; the child has ever been to prison or been on probation at 16 y.

4. Parental separation: The child has been separated from their father or mother due to death, divorce, or separation at 7, 11 or 16 y.

5. Mental illness: Household has contact with mental health services at 7 or 11 y; Family member has mental illness at 7 & 11 or 16 y.

6. Alcohol abuse: Family member has alcohol abuse problem at 7 y.

Exposure to adversity was identified by a positive response to any of the above categories. Respondents were excluded if they had missing data for all six categories. Respondents were considered as having no adversities if they answered ‘no’ all the categories or if they answered ‘no’ to one or more category and the other categories were missing. ACE was measured by counting the reports of: child in care, physical neglect, offenders, parental separation, mental illness and alcohol abuse. A three category variable was then constructed (0 adversities/1 adversity/2or + adversities).

### Early life socioeconomic and biological confounders

To examine the relationship between ACE and cancer, prior confounding variables potentially associated with both ACE and cancer need to be included in the initial multivariate model. Among the variables available at baseline, collected from the cohort member’s mothers via a questionnaire at birth, we identified those most likely to be social or biological confounding factors based on the literature. Household and parental characteristics were included: mother’s age at birth, overcrowding (people per room), mother’s partner’s social class (recoded into manual vs non-manual), and if this was unavailable the mother’s father’s social class was used mother’s education level (left school before/after minimum leaving age), and maternal smoking during pregnancy (no smoking, sometimes, often, heavy). The respondent’s characteristics and birth variables were also included: sex, gestational age at birth, parity, birth weight, foetal distress, problems during pregnancy and breastfeeding (no, one month or less, more than one month). To control for health problems in childhood, a childhood pathologies variable was constructed using data collected at ages 7, 11 and 16 y. It was based both on mother’s report and medical examinations including congenital conditions, moderate/severe disabilities, chronic respiratory or circulatory conditions, sensory impairments and special schooling (childhood pathology: yes/no).

### Mediators across the lifecourse

To determine whether any observed associations between ACE and cancer were due to adult mediating factors, the following were added to the models: respondent’s educational attainment at 23y (A level/ O level/ no qualification), respondent’s occupational social class at 23 y (non-manual active/ manual active/ inactive). The ‘malaise inventory’ was used to identify symptoms of depression at the age of 23. It was based on a set of 24 questions indentifying symptoms, if the respondent reported experiencing more than seven of the symptoms they were considered as having psychological malaise (no malaise/ malaise), characterized by symptoms of depression and/or anxiety. The health behaviour variables included were: alcohol consumption at 23 y (normal drinking (women: between 1–14 units in the previous week, men: between 1–21 units in the previous week)/ abstinence (reported not consuming any alcohol in the previous week)/ heavy drinking (women: >14 units in the previous week, men: >21 units in the previous week [28]), smoking status at 23 y (never smoked/ past smoker/ current smoker), and BMI (kg/m^2^) categorised using the WHO cut-offs age 23 y [29]. Adult life-style variables are available at other points along the lifecourse, however in our models, these adult variables at the age of 23 are a proxy for behavioural patterns in early adulthood predating reports of cancer. Controlling for them serves as a first step to understanding possible mechanisms. In the women’s model we also created a variable identifying the age at which cohort members had their first pregnancy, a known risk factor for breast cancer. This was determined based on variables at age 33 y, and 50 y. The resulting variable is in three categories (before 33 y/ after 33 y/ no information on pregnancy).

### Missing data, imputation and statistical analysis

**Figure 1:**
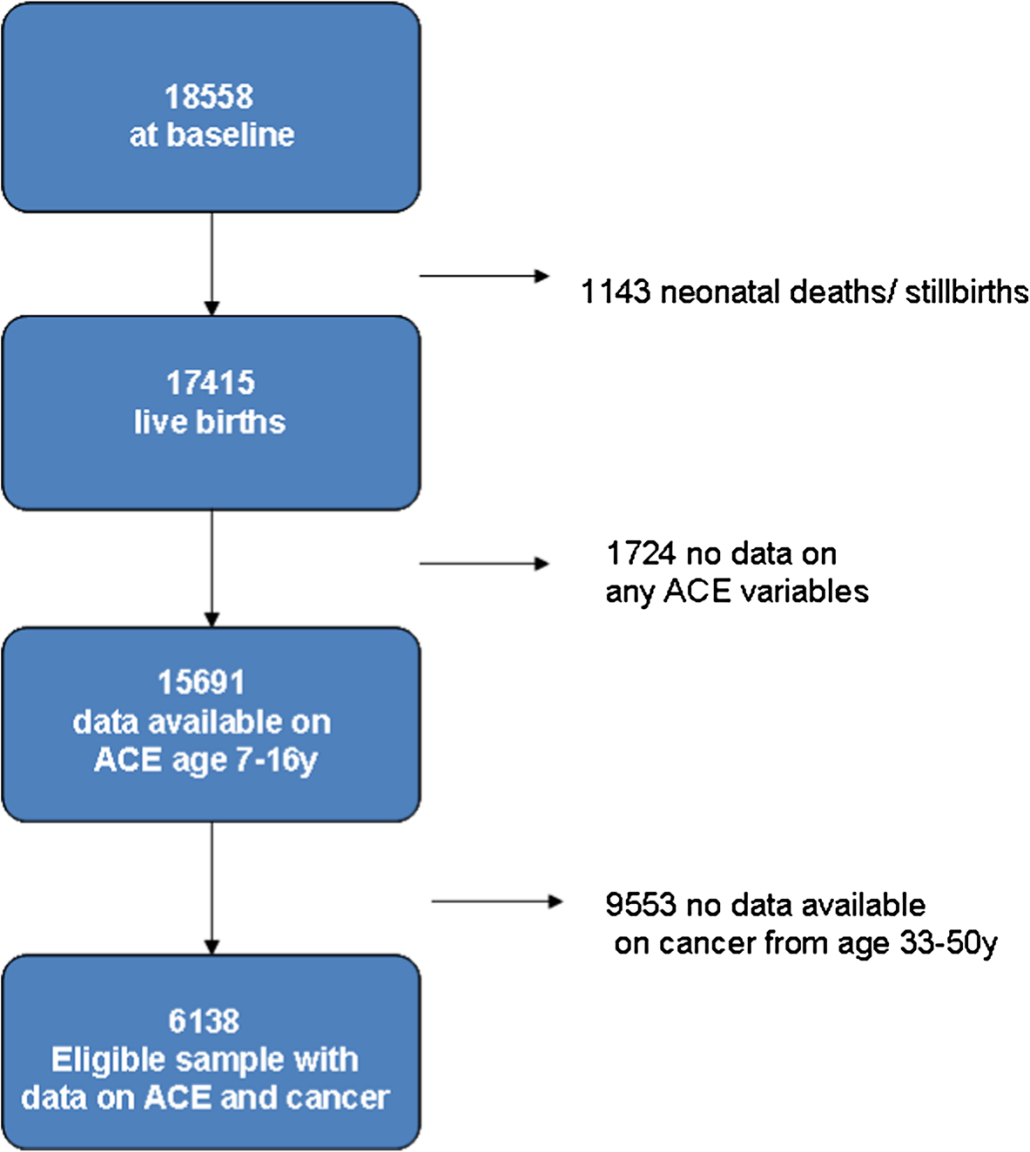
Flowchart showing selection of the subsample used for this study.

Cohort follow-up has been good over time, 84% participated at the age of 16, with a gradual dwindling in participation throughout adulthood (72% at age 23, and 65% at age 42) when participants were most likely to have moved [23]. Refusal rates have been low, with 7% at age 23 and 13% at age 42 for example [23]. In the latest data sweep (2008) 54% (n = 9790) of the original sample participated, and for some variables missing data presents a considerable challenge. The sample used for this study is described in Figure 1.

To control for possible bias due to missing data, we imputed data for covariates with missing data using the multiple imputation program ICE in STATA v11. Twenty imputations were conducted taking the missing at random (MAR) assumption for the covariates only (mother’s education, father’s social class, overcrowding, birthweight, gestational age, parity, smoking during pregnancy, mother’s age at birth, breastfeeding, child pathologies, educational attainment at 23, social class at 23, malaise inventory at 23, drinking at age 23 and smoking at age 23, age at 1^st^ pregnancy for women). Neither the exposure variable of interest (ACE) nor the cancer variable were included in the multivariate imputation model.

Bivariate crosstabulations were carried out on the imputed data using logistic regression to obtain p-values adjusted for the artificially inflated sample size. As the timing of cancer events was unknown, multivariate logistic regression analyses were carried out on the data obtained from multiply imputed data. Three models were run separately by sex, entering the variables chronologically as they would occur over the lifecourse. First early life socioeconomic circumstances and perinatal variables were entered. In model 2, childhood pathologies and ACE were added to the model. Finally, model 3 additionally controlled for education, social class, psychological malaise, health behaviours at 23 y, and for women, age at first pregnancy.

## Results

Descriptive statistics are presented in Table 1 for the subsample (n = 6138) who provided information on whether or not they had had a cancer in the last four data sweeps (ages 33, 43, 46 and 50). Among respondents who answered questions on cancer, 3.3% of men and 10.6% of women reported having had cancer by the age of 50. In total 444 self-reported cancers were identified in the cohort reported between the ages of 33 and 50, 80% being reported by women (n = 351). Based on the available information on cancer types reported by the cohort members (see Additional file 1), breast and cervical cancers were most often reported (19% and 15% respectively), followed by skin cancers (11%). Nevertheless, most cancers remained ‘undefined ‘(39%) The majority of men (74%) and women (75%) had had no adverse childhood experiences, based on the prospectively collected information at ages 7, 11 and 16 y. The distribution of ACEs was similar for both sexes, with 5.6% of men and 5.4% of women having two or more ACEs.

**Table 1:** Characteristics of the subsample* at birth, during childhood and in adulthood

		**Men % (n)**	**Women % (n)**
		**46.2 (2836)**	**53.8 (3302)**
**Cancer**	*No*	96.7 (2743)	89.4 (2951)
	*Yes*	3.3 (93)	10.6 (351)
**ACE**	*0*	74.4 (2110)	75.2 (2483)
	*1*	20.0 (566)	19.4 (641)
	*2 or more*	5.6 (160)	5.4 (178)
**Mother’s education**	*Stayed at school after min age*	27.3 (773)	26.5 (875)
	*Left school at or before min age*	69.5 (1970)	70.0 (2310)
	*Missing*	3.3 (93)	3.5 (117)
**Parental social class**	*Non-manual*	30.9 (875)	27.2 (931)
	*Manual*	65.7 (1862)	68.0 (2246)
	*Missing*	3.5 (99)	3.8 (125)
**Overcrowded household**	*<1.5 people per room*	85.5 (2424)	83.4 (2775)
	≥*1.5 people per room*	9.1 (257)	11.3 (374)
	*Missing*	5.5 (155)	5.2 (173)
**Parity**	*Primiparous*	36.5 (1034)	37.6 (1242)
	*One*	32.2 (913)	28.6 (945)
	*Two or more*	28.6 (812)	30.7 (1013)
	*Missing*	2.7 (77)	3.1 (102)
**Fœtal distress**	*No*	87.4 (2478)	89.1 (2943)
	*Yes*	9.9 (281)	7.8 (256)
	*Missing*	2.7 (77)	3.1 (103)
**Problems during pregnancy**	*No*	72.7 (2063)	70.7 (2333)
	*Yes*	24.5 (695)	26.2 (865)
	*Missing*	2.8 (78)	3.2 (104)
**Smoking in pregnancy**	*No*	66.6 (1888)	65.6 (2165)
	*Sometimes*	5.4 (152)	5.0 (164)
	*Moderately*	13.8 (390)	14.3 (472)
	*Heavily*	10.4 (296)	11.0 (364)
	*Missing*	3.9 (110)	4.1 (137)
**Breastfeeding**	*Yes for more than 1 month*	44.6 (1264)	43.4 (1432)
	*Yes for up to one month*	22.3 (631)	23.1 (763)
	*No*	27.4 (776)	27.3 (901)
	*Missing*	5.8 (165)	6.2 (206)
**Childhood pathologies**	*No*	65.0 (1843)	69.4 (2291)
	*Yes*	22.6 (640)	18.5 (612)
	*Missing*	12.5 (353)	12.1 (399)
**Education level 23 y**	*A levels or higher*	25.6 (726)	24.3 (801)
	*O levels*	39.4 (1117)	45.1 (1489)
	*No qualifications*	34.2 ((970)	29.1 (961)
	*missing*	0.8 (23)	1.5 (51)
**Social class 23 y**	*Non-manual active*	38.6 (1094)	54.3 (1792)
	*Manual active*	45.4 (1287)	12.8 (422)
	*Inactive*	12.9 (365)	31.1 (1027)
	*Missing*	3.17 (90)	1.9 (61)
**Alcohol consumption 23 y**	*Normal*	70.1 (1988)	56.4 (1862)
	*Abstinence*	4.2 (118)	7.4 (244)
	*Heavy*	17.6 (498)	8.9 (293)
	*missing*	8.2 (232)	27.4 (903)
**Smoking 23 y**	*Never*	29.3 (832)	33.4 (1102)
	*Past*	33.9 (960)	27.9 (921)
	*Current*	36.1 (1023)	37.3 (1230)
	*missing*	0.7 (21)	1.5 (49)
**Psychological malaise 23 y**	*No*	96.1 (2725)	89.5 (2956)
	*Yes*	3.0 (85)	8.9 (295)
	*Missing*	0.9 (26)	1.5 (51)
**Body mass index 23 y**	*Normal*	75.3 (2136)	76.5 (2526)
(Kg/m^2^ WHO cut-offs)	*Underweight*	2.3 (64)	7.0 (231)
	*Overweight*	16.8 (477)	11.3 (374)
	*Obese*	3.9 (110)	2.6 (86)
	*missing*	1.7 (49)	2.6 (85)
		**Mean (s.e.)**	**Mean (s.e.)**
**Gestational age in days**	*(missing n = 2986 )*	279.9 (0.2)	280.4 (0.2)
**Birthweight Kg**	*(missing n = 1775)*	3.4 (0.0)	3.2 (0.1)
**Mother’s age at birth**	*(missing n = 1156)*	27.5 (0.1)	27.6 (0.1)

*Subsample of individuals who provided information used to create adversity and cancer variables.

Table 2 shows bivariate analyses between ACE, the covariates and cancer for men and women. In men, no relationship is apparent between ACE and cancer. The multivariate models for men (Table 3) show no association between early life socioeconomic variables or perinatal variables and cancer. ACE was not significantly associated with cancer, and neither were social variables at age 23 y, or smoking and alcohol age 23 y.

**Table 2:** Descriptive statistics on ACE, and the lifecourse covariates by reported cancer for men and women

		**Men**		**Women**	
**% (n)**		**Cancer ‘yes’ % (N = 93)**	P*	**Cancer ‘yes’ % (n = 351)**	P*
**Cancer**		3.3		10.6	
**Characteristics in terms of reported cancer**					
**ACE**	*0*	3.1		9.1	
	*1*	3.5	0.586	13.0	0.004
	*2 or more*	5.0	0.189	23.0	<0.0001
**Mother’s education**	*Stayed at school after min age*	2.6		9.1	
	*Left school at or before min age*	3.6	0.112	11.2	0.058
**Parental social class**	*Non-manual*	2.7		8.6	
	*Manual*	3.6	0.176	11.5	0.010
**Overcrowded household**	*<1.5 people per room*	3.1		10.0	
	≥*1.5 people per room*	4.9	0.251	15.4	0.002
**Parity**	*Primiparous*	3.9		10.0	
	*One*	2.5	0.151	10.5	0.588
	*Two or more*	3.3	0.320	11.5	0.261
**Fœtal distress**	*No*	3.2		10.5	
	*Yes*	4.2	0.561	12.0	0.591
**Problems during pregnancy**	*No*	3.5		10.2	
	*Yes*	2.6	0.171	11.9	0.212
**Smoking in pregnancy**	*No*	3.1		10.0	
	*Sometimes*	5.2	0.039	11.6	0.458
	*Moderately*	4.6	0.045	11.2	0.300
	*Heavily*	1.8	0.215	13.5	0.072
**Breastfeeding**	*Yes for more than 1 month*	3.2		9.7	
	*Yes for up to one month*	3.4	0.884	11.1	0.275
	*No*	3.4	0.934	11.7	0.161
**Childhood pathologies**	*No*	3.4		9.9	
	*Yes*	3.0	0.411	13.3	0.016
**Education level 23 y**	*A levels or higher*	2.6		8.3	
	*O levels*	3.0	0.628	9.2	0.209
	*No qualifications*	4.0	0.113	14.6	<0.0001
**Social class 23 y**	*Non-manual active*	2.9		9.4	
	*Manual active*	3.4	0.320	11.5	0.222
	*Inactive*	3.6	0.628	12.2	0.018
**Alcohol consumption 23 y**	*Normal*	3.1		10.0	
	*Abstinence*	4.1	0.373	10.9	0.578
	*Heavy*	3.6	0.820	14.1	0.031
**Smoking 23 y**	*Never*	3.0		7.2	
	*Past*	3.0	0.797	9.2	0.214
	*Current*	3.7	0.504	14.6	<0.0001
**Psychological malaise 23 y**	*No*	3.2		10.0	
	*Yes*	3.4	0.999	16.8	0.001
**Body mass index 23 y**	*Normal*	2.7		10.4	
(Kg/m^2^ WHO cut-offs)	*Underweight*	4.2	0.703	13.9	0.092
	*Overweight*	4.5	0.159	10.7	0.662
	*Obese*	7.3	0.036	6.1	0.285
**Age at first pregnancy**	*Before 33*			9.9	
	*After 33*			18.4	<0.0001
	*No information on pregnancy*			8.4	0.225

*p-values comparing ‘yes’ v ‘no’ cancers for each covariate.

**Table 3:** Multivariate logistic regression models using data obtained from multiple imputation: men (n = 2836)

		**Model 1 early life factors**		**Model 2 + childhood pathologies and ACEs**		**Model 3 + adult mediating factors**	
		**OR (95% CI)**	**p**	**OR (95% CI)**	**p**	**OR (95% CI)**	**p**
**Mother's education**	*Left school after min age*						
	*Left school at or before min age*	1.26 (0.73-2.17)	0.412	1.25 (0.72-2.16)	0.427	1.18 (0.67-2.09)	0.563
**Father’s social class**	*Non-manual*						
	*Manual*	1.16 (0.68-1.96)	0.587	1.14 (0.67-1.94)	0.620	1.07 (0.62-1.86)	0.810
**Overcrowded household**	*<1.5 people per room*						
	*> = 1.5 people per room*	1.43 (0.76-2.68)	0.265	1.40 (0.75-2.64)	0.295	1.36 (0.72-2.59)	0.344
**Gestational age**	*(days)*	1.01 (0.99-1.03)	0.444	1.01 (0.99-1.03)	0.445	1.01 (0.99-1.03)	0.444
**Parity**	*Primiparous*						
	*One*	0.73 (0.42-1.26)	0.263	0.72 (0.42-1.25)	0.245	0.71 (0.41-1.23)	0.215
	*Two or more*	0.95 (0.52-1.73)	0.855	0.90 (0.48-1.66)	0.726	0.85 (0.45-1.60)	0.613
**Birthweight**	*(kg)*	0.77 (0.49-1.21)	0.259	0.78 (0.49-1.23)	0.279	0.75 (0.47-1.20)	0.231
**Fœtal distress**	*No*						
	*Yes*	1.38 (0.72-2.65)	0.336	1.37 (0.71-2.63)	0.348	1.35 (0.70-2.6)	0.376
**Problems during pregnancy**	*No*						
	*Yes*	0.69 (0.41-1.18)	0.175	0.69 (0.41-1.18)	0.180	0.71 (0.42-1.21)	0.210
**Smoking during pregnancy**	*No*						
	*Sometimes*	1.62 (0.75-3.49)	0.222	1.59 (0.74-3.44)	0.238	1.52 (0.70-3.33)	0.294
	*Moderately*	1.38 (0.8-2.37)	0.250	1.36 (0.79-2.35)	0.270	1.36 (0.78-2.36)	0.281
	*Heavily*	0.54 (0.21-1.37)	0.194	0.52 (0.2-1.34)	0.176	0.50 (0.20-1.28)	0.149
**Mother’s age at birth**	*(years)*	0.99 (0.95-1.04)	0.731	0.99 (0.95-1.04)	0.809	1.00 (0.95-1.04)	0.887
**Breastfed**	*Yes, for more than 1 month*						
	*Yes, for up to one month*	1.03 (0.58-1.81)	0.924	1.04 (0.59-1.82)	0.905	1.04 (0.59-1.84)	0.898
	*No*	1.00 (0.59-1.68)	0.991	0.99 (0.59-1.68)	0.982	0.96 (0.56-1.64)	0.885
**Childhood pathologies**	*No*						
	*Yes*			0.87 (0.51-1.49)	0.623	0.83 (0.48-1.43)	0.500
**ACE**	*No adversities*						
	*One adversity*			1.11 (0.66-1.87)	0.687	1.03 (0.61-1.76)	0.904
	*Two or more adversities*			1.50 (0.68-3.29)	0.314	1.39 (0.62-3.11)	0.419
**Education level at 23 y**	*A levels or higher*						
	*O levels*					1.00 (0.51-1.96)	0.995
	*No qualifications*					1.24 (0.56-2.73)	0.599
**Social class 23 y**	*Non-manual active*						
	*manual active*					0.92 (0.5-1.69)	0.788
	*Inactive*					1.06 (0.50-2.24)	0.885
**Smoking 23 y**	*Never*						
	*Past*					1.02 (0.55-1.87)	0.962
	*Current*					1.21 (0.68-2.18)	0.516
**Alcohol 23 y**	*Normal*						
	*Abstainance*					1.33 (0.49-3.59)	0.573
	*Heavy*					1.10 (0.62-1.94)	0.743
**Psychological malaise 23 y**	*No*						
	*Yes*					0.95 (0.23-3.92)	0.938
**BMI groups**	*Normal*						
	*Underweight*					1.40 (0.33-5.89)	0.647
	*Overweight*					1.58 (0.90-2.78)	0.113
	*Obese*					**2.72 (1.17-6.32)**	**0.020**

In women (Table 2), a bivariate relationship between ACE and cancer was shown. The proportion of women reporting a cancer before 50 increased from 9.1% for those with no ACEs to 13.0% for those with one ACE (p = 0.004), and 23.0% for those with 2 + ACEs (p < 0.001). Both manual parental social class and overcrowding in the household at birth were significantly related to reporting a cancer before 50 y, and mother’s education was of borderline significance. Women who had experienced childhood pathologies were also more likely to have had a cancer than those without childhood pathologies (13.3% vs 9.9%, p = 0.016). Many of the female respondent’s adult variables were also related to having had a cancer before 50 y in the bivariate analyses. Low educational attainment, being inactive in terms of occupation, being a heavy alcohol drinker, being a smoker and having symptoms of depression/ anxiety, and having a first pregnancy after the age of 33 y, were also related to having had a cancer before 50.

The multivariate models for women (Table 4) show that. ACE was significantly associated with reporting cancer before the age of 50 after control for potential confounding variables (model 2). The graded association showed that women with one ACE had a 40% increase in the odds of reporting cancer (p = 0.016), and those with two or more ACEs had a 2.5 increase in the odds of reporting a cancer versus women with no ACEs (p < 0.001). When information on adult behaviours, social characteristics and psychological malaise was added to the model this association weakened, so that women with two or more ACEs had a 2.1 increase in the odds of reporting a cancer versus those with no ACEs (p < 0.001). Women who had their first pregnancy after the age of 33 y had a significantly increased risk of having cancer before 50 y (p < 0.001). Furthermore, women who were smokers at age 23 y had a 70% increase in the odds of having cancer before 50 versus non-smokers (p = 0.001), and women who were heavy drinkers at age 23 y also had an increased risk of having cancer before 50, however this result was of borderline statistical significance (p = 0.055). Having symptoms of depression/anxiety increased the odds of having a cancer before 50 by 35%, however this result was not significant at the 5% level (p = 0.1).

**Table 4:** Multivariate logistic regression models using data obtained from multiple imputation: women (n = 3302)

		**Model 1 early life factors**		**Model 2 + childhood pathologies and ACEs**		**Model 3 + adult mediating factors**	
		**OR (95% CI)**	**p**	**OR (95% CI)**	**p**	**OR (95% CI)**	**p**
**Mother’s education**	*Left school after min age*						
	*Left school at or before min age*	1.06 (0.80-1.41)	0.681	1.06 (0.79-1.41)	0.697	1.07 (0.79-1.45)	0.659
**Father’s social class**	*Non-manual*						
	*Manual*	1.17 (0.88-1.56)	0.286	1.11 (0.83-1.49)	0.473	1.13 (0.84-1.53)	0.419
**Overcrowded household**	*<1.5 people per room*						
	*> = 1.5 people per room*	**1.47 (1.07-2.04)**	**0.019**	1.39 (1.0-1.92)	0.051	1.33 (0.95-1.85)	0.093
**Gestational age**	*(days)*	1.00 (0.99-1.01)	0.648	1.00 (0.99-1.01)	0.664	1.00 (0.99-1.01)	0.889
**Parity**	*Primiparous*						
	*One*	1.17 (0.88-1.57)	0.283	1.14 (0.85-1.52)	0.391	1.06 (0.78-1.43)	0.715
	*Two or more*	1.32 (0.95-1.83)	0.095	1.20 (0.86-1.67)	0.279	1.09 (0.77-1.54)	0.642
**Birthweight**	*(kg)*	0.90 (0.70-1.16)	0.425	0.91 (0.71-1.18)	0.488	0.93 (0.72-1.21)	0.585
**Fœtal distress**	*No*						
	*Yes*	1.24 (0.81-1.89)	0.316	1.25 (0.82-1.90)	0.306	1.23 (0.81-1.89)	0.334
**Problems during pregnancy**	*No*						
	*Yes*	1.16 (0.91-1.49)	0.240	1.13 (0.88-1.45)	0.332	1.14 (0.88-1.47)	0.324
**Smoking during pregnancy**	*No*						
	*Sometimes*	1.08 (0.65-1.79)	0.771	0.94 (0.56-1.57)	0.801	0.91 (0.54-1.54)	0.739
	*Moderately*	1.07 (0.78-1.49)	0.664	1.06 (0.76-1.46)	0.742	1.00 (0.72-1.39)	0.997
	*Heavily*	1.30 (0.93-1.82)	0.126	1.22 (0.87-1.72)	0.251	1.17 (0.82-1.66)	0.384
**Mother’s age at birth**	*(years)*	**0.98 (0.95-1.00)**	**0.040**	0.98 (0.96-1.00)	0.086	0.98 (0.96-1.01)	0.176
**Breastfed**	*Yes, for more than 1 month*						
	*Yes, for up to one month*	1.10 (0.83-1.46)	0.517	1.09 (0.81-1.45)	0.567	1.08 (0.80-1.45)	0.627
	*No*	1.14 (0.87-1.51)	0.340	1.09 (0.82-1.44)	0.550	1.08 (0.81-1.43)	0.601
**Childhood pathologies**	*No*						
	*Yes*			1.31 (0.99-1.73)	0.061	1.24 (0.93-1.65)	0.149
**ACE**	*No adversities*						
	*One adversity*			**1.40 (1.06-1.83)**	**0.016**	1.30 (0.98-1.72)	0.066
	*Two or more adversities*			**2.46 (1.66-3.65)**	**<0.001**	**2.14 (1.42-3.21)**	**<0.001**
**Age at 1st pregnancy**	*<=33y*						
	*>34y*					**2.24 (1.63-3.08)**	**<0.001**
	*No pregnancies, or no information*					0.94 (0.65-1.34)	0.716
**Education level at 23 y**	*A levels or higher*						
	*O levels*					0.95 (0.67-1.34)	0.756
	*No qualifications*					1.30 (0.87-1.93)	0.198
**Social class 23 y**	*Non-manual active*						
	*manual active*					0.93 (0.64-1.37)	0.726
	*Inactive*					1.04 (0.77-1.41)	0.796
**Smoking 23 y**	*Never*						
	*Past*					1.27 (0.9-1.78)	0.174
	*Current*					**1.72 (1.25-2.36)**	**0.001**
**Alcohol 23 y**	*Normal*						
	*Abstainance*					1.00 (0.66-1.52)	0.983
	*Heavy*					1.42 (0.99-2.04)	0.055
**Psychological malaise 23 y**	*No*						
	*Yes*					1.35 (0.94-1.93)	0.099
**BMI groups**	*Normal*						
	*Underweight*					1.26 (0.81-1.95)	0.299
	*Overweight*					0.93 (0.63-1.37)	0.706
	*Obese*					0.47 (0.17-1.24)	0.126

## Discussion

The main finding from this study was that psychosocial adversity in childhood was related to cancer incidence before 50 y among women, after adjusting for prior confounding factors and potential mediators, in a large prospective cohort. An accumulation of ACE remained a strong predictor of cancer in women, after taking important potential mediating factors at age 23 y into account, including smoking and drinking. Women who experienced two or more ACE doubled their risk of having a cancer before 50 relative to women who had had no childhood adversities. There was a tendency towards a graded association between childhood adversity and adult cancer across the ACE categories. These results make a significant contribution to demonstrating and understanding links between ACE and cancer because they use an a priori definition of adversity and identify ACE with prospectively collected data. The strength of the relationship between adversity and cancer was of the same magnitude as that observed between age at first pregnancy and cancer, a well-known risk factor for breast cancer [30].

The two main weaknesses of this study are in the self-reported nature of cancer incidence, and the amount of missing data caused by attrition in the cohort study. These weaknesses are partly addressed by the conservative nature of our cancer variable, and by using estimates obtained from multiple imputations to account for the missing data. We conducted sensitivity analyses by running the model using imputed datasets, using a case–control sub-sample, and using the full cohort dataset without imputation, and found that the relationship between ACE and cancer incidence was stable. Comparisons were made between complete-case analyses and those run on estimates obtained by imputation. The models yielded similar results until the inclusion of variables at age 23 (model 3). The differences observed in the results for model 3 indicate selection bias in the complete case sample, where individuals who had experienced ACEs in childhood were more likely to have missing data at age 23 regarding health behaviours. The multiple imputation model therefore enables adjustment for this bias. We are confident, therefore, that our analyses show a ‘real’ association, the nature of which needs to be established in further more complex modelling of mediating factors.

The nature of the questions collecting information on cancer varied by data sweep, some being retrospective (have you *ever* had cancer) while other questions asked about suffering from an illness at the time of the survey. This means that there are information gaps, notably between the age of 33 and 42 where the cohort member could have had cancer and not report that they were suffering from it subsequently. However, this means that our cancer variable is most likely conservative. The validity of self-reported cancer is likely to vary based on the cancer type diagnosed, and tends towards an underreporting of cancers, given the high levels of specificity reported in studies comparing self-reports to registry data [31]. Indeed in studies where cancer registry data have been compared to self-reported information individuals tend to underreport rather than overreport cancer history, and variation in inaccurate cancer reporting varied considerably by cancer type [32]. It is also important to consider the biases linked with overreporting cancers. Individuals whose self-reported cancer was unconfirmed by the Finnish cancer registry were more likely to have accumulated psychosocial strain across their lifecourse [33]. The probability of misreporting cancer, both over-reporting and under-reporting, has been associated with socio-demographic characteristics: gender, age, BMI, size of household, place of birth, smoking, social participation, educational level, type of employment, alcohol consumption and poor well-being [34]. The sensitivity of self-reported cancer is also likely to be higher among respondents with a high level of education [31]. Stavrou et al. have demonstrated a good level of sensitivity and specificity for self-reported cancer diagnoses in a cohort of older women in Australia, comparing self-reports to registry data [35]. Gupta et al. also describe good concordance between self-reported cancer and medical chart information [36]. The multivariate models also show associations between well known risk factors and cancer before 50, such as age at first pregnancy, smoking and drinking, which contributes to enhancing the validity of the cancer variable.

Women were four times more likely to report having had a cancer than men which is understandable given the age of cohort participants at the time of study. Based on 2007–2009 estimates of cancer incidence in the UK, 44% of cancers diagnosed among women aged 25–49 were breast cancers [37]. Rather than indicating a sex, or gender difference, the lack of association between virtually any of the variables and cancer among men is likely be mainly due to the far lower incidence in men up to age 50 (93 cancers). The distribution of cancer types observed in the cohort will continue to evolve over time, and begin to represent a greater proportion of men due to the increased occurrence of prostate and lung cancer among men >50 years. Linkage will hopefully be made between the cohort and the cancer registries which would significantly increased the validity and reliability of the information, and allow analyses by cancer type in the future, and survival analyses, which are currently not possible due to a lack of information on the timing of events. For these analyses we limited the health behavioural variables to those collected at age 23 rather than using variables available afterwards. This enabled to limit any further problems of missing data, but may lead to an underestimate of the contribution of health behaviours in the associations.

One important strength of this study is in the prospective nature of the information on ACEs. Most studies examining the links between early adversity rely on retrospective questions, which are prone to recall bias, an important issue raised by Korpimaki et al. using data from a working age population and the cancer registry [19]. Using a retrospective questionnaire to identify ACE, and limiting their analyses to individuals whose cancer was diagnosed subsequent to answering questions on ACE to address the problem of recall bias, these authors found no association between working-age cancer and reporting ACE.

There is currently insufficient power to work on cancers at different sites using the cohort. Furthermore, we take a lifecourse approach to this study and are interested in the pre-cancerous phase where early psychosocial adversity may increase susceptibility to developing cancer earlier. The cause of cancers and their prognoses are indeed diverse, however our aim here is to understand the factors contributing to susceptibility at its earliest phase. The ACE literature indicates that there are early life factors which may increase inflammatory response [38], decrease immune system efficiency [39], increase exposure to viral infections [40], and raise the probability of damaging health behaviours along the lifecourse [41,42]. All of these behavioural and biological processes are known to be involved in cancer development.

The association between stress and cancer development and progression has been shown in biological studies [5]. Stress-related immunological changes bring about declines in natural killer cell activity by depressing their ability to respond to tumour or virally infected cells, and causing a reduction in the body’s defences linked to the repair of damaged DNA [43]. Exposure to stressors is known to trigger responses via the central nervous system produced by the hypothalamic-pituitary-adrenal axis (HPA). This activity modifies neuroendocrine pathways which, over the long term, alter the critical physiological mechanisms involved in tumourogenesis [5]. When exposed to chronic stress, “the body remains in a constant state of overdrive” with adverse consequences on the regulation of systems implicated in cancer progression [6]. ACE has also been associated with risky health behaviours such as smoking, alcoholism, early sexual activity, and having multiple sexual partners [8,13,38-41], all of which are ‘indirect’ risk factors for cancer.

Our hypothesis that adversity in childhood may be linked with biological susceptibility involved in the development of cancers in adult life is reinforced by our findings. It could operate via two main mechanisms: A direct biological effect and an indirect effect via health behaviours (or a combination of both). The fact that ACE is still associated with cancer even after adjusting for behavioural and social mediators favours argument for a direct “biological” role of ACE.

In epidemiological studies, evidence of a direct association between exposure to stress and cancer incidence is mixed and inconclusive. This is likely to be due to the different ways in which stress was conceptualised. A Danish cohort study on 8736 men and women found no direct association between cumulative stressful life events collected retrospectively, mostly during adulthood, and cancer incidence, though they did identify a relationship between stress and unhealthy lifestyles [44]. Ollonen (2005) et al. found support for an overall association between stressful life events across the lifecourse and breast cancer risk in their Finnish case–control study [45]. A meta-analysis of studies on the association between stress and breast cancer did not support an association between stressful life events in adulthood and breast cancer risk [46]. Using linkage to the cancer registry, Fang et al. found that bereaved parents were at increased risk for cancers with an infectious aetiology, especially those linked to infection by the Human Papilloma Virus (HPV) after controlling for confounders. The authors hypothesised that the stress induced by losing a child may accelerate the cancer genesis of an established infection [47]. These studies do not consider the timing of exposure to stressful events in their analyses, an important factor considering the differential effects of physiological stress responses on various areas of the brain depending on when exposure occurs along the lifecourse [48].

## Conclusion

Our findings establish an association between ACE and cancer in women using prospective data. The suggestion that cancer risk may be influenced by conditions in the first years of life is potentially important in furthering our understanding of factors contributing to cancer development, and consequently in redirecting scientific research and developing appropriate prevention policies. This nevertheless remains a first step in understanding the relationship between ACE and cancer in the 1958 birth cohort. A second step will require an in-depth exploration of both direct and indirect pathways along which biological, social and psychosocial mechanisms are likely to operate.

## Abbreviations

ACE: Adverse childhood experiences; NCDS: National child development study; HPA: Hypothalamic-pituitary-adrenal axis.

## Competing interests

The authors declare that they have no competing interests.

## Authors’ contributions

MKI was involved in the conception and design of the study, analysing and interpreting the data, drafted the manuscript and made modifications. PG, CD, and TL were involved in the conception and design of the study, analysing and interpreting the data, and revising the manuscript. BL and DD analysed and interpreted the analyses, and revised the manuscript. RL, NC, MB, and DB interpreted the analyses and revised the manuscript. The first author had full access to all of the data in the study and takes full responsibility for the integrity of the data and the accuracy of the data analysis. All authors approved the current version.

## Pre-publication history

The pre-publication history for this paper can be accessed here:

http://www.biomedcentral.com/1471-2458/13/767/prepub

## Supplementary Material

Additional file 1 Self-reported cancer types in the NCDS between age 33 and 50 years. Click here for file
