# Supplementary material for: Childhood adversity as a risk for cancer: findings from the 1958 British birth cohort study
Source: BMC Public Health. 2013 Aug 19;13:767. doi: 10.1186/1471-2458-13-767 (PMC3765119; doi:10.1186/1471-2458-13-767)
Supplement: Additional file 1 — Self-reported cancer types in the NCDS between age 33 and 50 years. [file 1471-2458-13-767-S1.doc]

Supplementary Table : Self-reported and recoded cancer types in the NCDS between age 33 and 50

| **Cancer types identified** | **Cumulative number of cancer types identified age 33-50** | **Cumulative % of cancer types** |
| --- | --- | --- |
| Bone | 4 | 0.7 |
| Brain | 3 | 0.5 |
| Breast | 111 | 18.5 |
| Cervical | 91 | 15.2 |
| Colon | 16 | 2.7 |
| Hodgkin's lymphoma | 19 | 3.2 |
| Kidney | 1 | 0.2 |
| Leukaemia | 15 | 2.5 |
| Liver | 3 | 0.5 |
| Lung | 3 | 0.5 |
| Mouth, lips, larynx, pharynx | 5 | 0.8 |
| Non-Hodgkin's lymphoma | 7 | 1.2 |
| Other organs | 1 | 0.2 |
| Ovaries | 1 | 0.2 |
| Pancreas | 1 | 0.2 |
| Prostate | 6 | 1.0 |
| Skin | 63 | 10.5 |
| Stomach | 2 | 0.3 |
| Testicular | 16 | 2.7 |
| Thyroid | 5 | 0.8 |
| Undefined | 209 | 34.8 |
| Uterus | 14 | 2.3 |
| Other | 4 | 0.7 |
| Total | 600 | 100.0 |
| Duplicates & multiple cancers | 156 | 26.0 |
| Total cancers identified | 444 | 74.0 |
